# Optic flow, a rich source of optic information for harbour seals (*Phoca vitulina*)

**DOI:** 10.1242/jeb.250168

**Published:** 2025-05-29

**Authors:** Laura-Marie Sandow, Ann-Kathrin Thimian, Markus Lappe, Frederike D. Hanke

**Affiliations:** ^1^ University of Rostock, Institute for Biosciences, Neuroethology, 18059 Rostock, Germany; ^2^ University of Münster, Institute for Psychology, 48149 Münster, Germany

**Keywords:** Marine mammal, Motion vision, Vision, Heading, Movement

## Abstract

Marine mammal vision is often considered to only provide limited information, particularly underwater in low light levels and turbidity. However, when these animals move through turbid water optic flow is elicited. A past study has documented the harbour seal's (*Phoca vitulina*) ability to perceive deviations from heading from optic flow simulating movement through a volume of turbid water. Here, we asked whether harbour seals are also able to perceive and analyse surface optic flow. Thus, we simulated three optic flow environments and trained three harbour seals to determine the simulated heading. The harbour seals precisely indicated their heading with a mean (±s.d.) accuracy of 4.61±0.56 deg for volume optic flow, 4.96±0.74 deg for surface optic flow mimicking movement over a surface and 3.58±1.12 deg for surface optic flow mimicking movement underneath a surface. We conclude that harbour seals have access to and can thus rely on optic (flow) information whenever there is enough light for vision, thus refuting existing opinions about poor visual guidance in harbour seals or, more generally, in marine mammals. A detailed analysis of optic flow perception in (semi-) aquatic animals is expected to enhance our understanding of optic flow perception and vision in general.

## Teaser

**Highlighted Article:** Harbour seals precisely determine the direction of heading from optic flow simulations. Optic flow perception generally provides seals with access to a wealth of optic information valuable for visually guided behaviours.

## INTRODUCTION

Living in water often challenges its inhabitants with poor viewing conditions. These conditions are caused by low light levels and turbidity, the latter originating from floating particles such as phytoplankton, zooplankton or whirled up grains of sand. Consequently, many researchers hypothesized that vision in aquatic animals is less important than other sensory systems for the guidance of complex behaviours such as foraging or navigation (e.g. for harbour seals Dehnhardt et al., 2014). However, even when viewing conditions deteriorate, for example as a result of a high level of turbidity, aquatic organisms that actively move through particle-rich water can get visual information as optic flow is generated. Optic flow is the motion pattern generated in the retina of the eye of an observer that is moving relative to the environment (Gibson, 1950). The point the animal is moving towards is called focus of expansion (FOE). Thus, the FOE indicates the animals' direction of self-motion, called heading, and can be used for the control of self-motion (Gibson, 1950; Warren, 2008). Aquatic animals could particularly benefit from optic flow when, for example, navigating the open ocean, in which, under many circumstances, few optic cues are available for the determination of their own position, direction or speed of movement besides optic flow information.

Optic flow information can inform numerous behaviours. Besides the determination of heading, or deviations from it, or generally the regulation of self-motion (Gibson, 1950; Warren, 2008), aquatic animals might profit from optic flow with respect to the determination of travelled distances (Esch and Burns, 1995; Ronacher and Wehner, 1995; for general distance estimation abilities of harbour seals see Maaß and Hanke, 2021), assessing time to collision with an obstacle (Sun et al., 1992) or swimming speed (Baird et al., 2011; Barron and Srinivasan, 2006). Additionally, information about an observer's distance to objects could be derived from optic flow.

Gläser et al. (2014) challenged the idea of optic flow perception in a semi-aquatic mammal, the harbour seal (*Phoca vitulina*), building on the first studies of the harbour seal's motion vision abilities (Hanke et al., 2008; Weiffen et al., 2014). The experimental animal of Gläser et al.’s (2014) optic flow study was able to perceive optic flow that mimicked movement through a cloud of particles. More specifically, the harbour seal was able to indicate the deviation of a superimposed cross from the simulated heading, the FOE, with an accuracy of 0.6 deg. In conclusion, contrary to previous thoughts of turbidity only having detrimental effects on vision, the results of Gläser et al. (2014) suggested that harbour seals gain a wealth of visual motion information when manoeuvring in turbid water. The previous documentation of a saccadic movement strategy in harbour seals (Geurten et al., 2017) furthermore highlights that harbour seals optimize the time during which distance estimation from optic flow is possible, namely during translational movements. Moving saccadically means that harbour seals minimize the duration of rotational movements, which do not provide any depth information (Koenderink, 1986; Koenderink and van Doorn, 1987), by doing short burst-like movements, called saccades, which occur between long periods of translational movements.

As a continuation of research into optic flow perception in harbour seals, the first aim of the present study was to test whether harbour seals are able to estimate the simulated heading from surface optic flow in addition to the previously tested volume optic flow (Gläser et al., 2014). Surface optic flow is elicited when harbour seals are swimming over the seabed, underneath the water surface or with their heads above the water surface. If harbour seals were able to perceive a simulated heading from surface and volume optic flow environments, they would permanently have access to optic flow information, provided enough light is available causing a perceivable contrast of at least a few objects to the environment. We assume conditions under which harbour seals experience the total absence of light and even absence of objects such as particles to rarely occur, if at all, as harbour seals usually stay close to shore where some light is expected to be present during the day, but also at night, in air and in the shallow coastal waters and as they have very sensitive eyes (for review, see Hanke et al., 2009). Thus, in our study, we presented three harbour seal individuals with volume optic flow and two scenarios of surface optic flow, one simulating movement over a surface, the other simulating movement underneath a surface.

In our study, we asked the harbour seals to determine from optic flow whether the simulated heading was to the left or right, i.e. we determined heading accuracy thresholds (HATs). HATs were not determined for harbour seals in Gläser et al.’s (2014) optic flow study, as instead the harbour seal's ability to determine deviations from the simulated heading was assessed. For the assessment of deviations, Gläser et al. (2014) presented a reference point, a cross, which was either congruent or not congruent with the FOE, and the harbour seal had to indicate whether the reference point was deviating or not. As, however, reference points might only be rarely available in the habitat of harbour seals, we changed the experimental approach in the current study to more closely mimic the conditions we anticipate harbour seals to encounter in their natural habitat when relying on optic flow information.

Overall, we were interested in two comparative aspects. First, we wanted to compare the accuracy of heading determination from optic flow elicited when moving through a volume versus moving above or underneath a surface. We thus determined heading accuracy in air from three optic flow scenarios in this study. We could not compare our results with the previously obtained results (Gläser et al., 2014) because of (1) the slightly changed experimental approach, as just mentioned, and (2) the different medium, in air (this study) versus in water (Gläser et al., 2014), in which optic flow perception was tested. The different medium might affect the accuracy of heading determination but not the general ability to perceive optic flow. Second, we were interested to compare our results obtained in harbour seals with those from other organisms. However, to our knowledge, optic flow studies involving comparable experimental approaches, obtained during behavioural experiments, have only been conducted with humans and primates (e.g. Warren et al., 1988; Warren and Hannon, 1990; Van den Berg and Brenner, 1994; Lappe et al., 1996; Britten and van Wezel, 2002). As comparisons across studies are generally difficult because of methodological differences, we carried out the experiment with all three optic flow environments with human participants under the same experimental conditions to relate optic flow perception in harbour seals to our own perception.

## MATERIALS AND METHODS

### Experimental subjects

#### Experimental animals

The experiment was conducted at the Marine Science Centre of the University of Rostock, Germany. Three male harbour seals, *Phoca vitulina* Linnaeus 1758, named ‘Nick’ (born in 1999), ‘Luca’ (born in 2002) and ‘Miro’ (born in 2019) were available for testing during the experimental period. They were housed in a large seawater enclosure in a mixed group of harbour seals, California sea lions (*Zalophus californianus*) and a South African fur seal (*Arctocephalus pusillus*). As they were kept outdoors, they experienced a natural rhythm of day and night. Nick and Luca were experimentally experienced and had already taken part in several scientific studies including two-alternative forced-choice tasks (e.g. Heinrich et al., 2016, 2020; Sandow and Hanke, 2024; Scholtyssek et al., 2008). Miro was naive with respect to scientific experiments.

The harbour seals were fed 1–4 kg of herring (*Clupea harengus*) or sprats (*Sprattus sprattus*) per day, depending on season and motivation. They received the majority of their daily food (1–2 kg) during the experiments, which were carried out 5–6 days a week.

The behavioural experiment carried out in this study was in accordance with the European Communities Council Directive of 22 September 2010 (2010/63/EU) and the German Animal Welfare Act of 2006. The individuals used in the study were not subjected to pain, suffering or injury; therefore, no approval or notification was required.

#### Human participants

Three female human participants aged between 24 and 36 voluntarily participated in this study. All participants had normal or corrected-to-normal vision with glasses or contact lenses. They were informed about the scientific background and the experimental procedure and provided consent that their age and sex could be recorded with the anonymized data.

We received approval from the ethics committee of the University Medicine Rostock for conducting the behavioural experiment with human participants (registration number: A 2024-0083).

### General experimental setup for the harbour seals

**Fig. 1.**
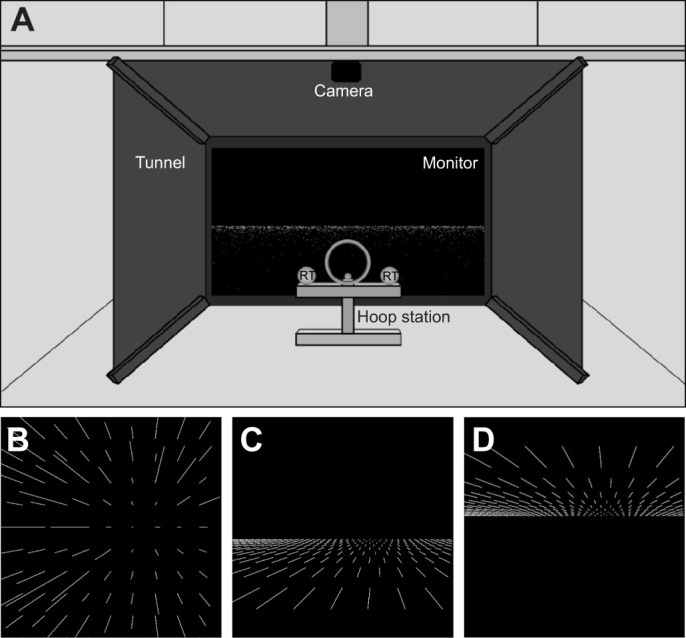
**Illustration of the experimental setup and stimuli used for testing aerial optic flow perception in harbour seals.** (A) Experimental setup for testing optic flow perception in harbour seals in air. A 42-inch monitor was installed in the experimental chamber. The monitor was surrounded by a tunnel of black velvet (100 cm wide, 65 cm high, 120 cm long) and served to present the stimuli (here: above-surface optic flow). With the help of the tunnel and because the setup was installed in an experimental chamber, we achieved a constant ambient illumination of <0.1 lx in the experimental area in front of the monitor. The tunnel also directed the harbour seals' attention towards the monitor. In front of the monitor, a metal hoop station was installed ensuring a constant distance of the harbour seals to the monitor. Additionally, to the left and right of the hoop, two response targets (RT) were installed to which the harbour seals responded in line with a two-alternative forced-choice experiment. The behaviour of the harbour seals was observed via a camera from the back of the chamber where the experimenter was hiding behind a curtain (not shown) out of the field of view of the harbour seals to avoid secondary cueing. (B) Schematic visualization of the presented optic flow field during simulation of a translational forward movement through a volume. (C) Schematic visualization of the presented optic flow field when simulating a translational forward movement above a surface. (D) Schematic visualization of the presented optic flow field when simulating a translational forward movement underneath a surface. For a detailed description of the stimuli see Materials and Methods, ‘Stimuli’. The lines indicate the associated velocity vectors. The focus of expansion (FOE) is always on the right side of the midline of the monitor.

As the experiment was performed in air, the experimental setup was mounted in an experimental chamber (2 m wide, 2 m high, 3 m long). Working in a chamber allowed us to achieve constant test conditions during the sessions. For stimulus presentation, a 42-inch monitor (Toshiba LCD Colour TV, Model no. 42XV632D, refresh rate 60 Hz, resolution 1920×1080, Toshiba Corporation, Tokyo, Japan) was installed in the chamber. It was surrounded by a tunnel of black velvet (100 cm wide×65 cm high×120 cm long; Fig. 1), to direct the harbour seals' focus completely onto the stimuli presented on the monitor and to achieve a constant illumination of <0.1 lx in the experimental area (Multifunctional Environment Measuring Instrument 4 in 1, Voltcraft, Hirschau, Germany). In front of the monitor, a metal hoop station was screwed into the ground. During trials, the harbour seals had to position their heads in the hoop, ensuring a constant head position relative to the monitor and a constant viewing distance of 60 cm to the monitor. From this distance, the monitor covered 76 deg of the visual field; thus, the simulations filled a larger part of the visual field than the binocular visual field of a harbour seal (Hanke et al., 2006). The height of the hoop station was individually adjusted to achieve an eye height 36 cm above ground for all harbour seals. This eye height ensured that the harbour seals' eyes were pointing towards the centre of the monitor. To the left and right side of the hoop, a response target was mounted, towards which the harbour seals directed their responses (Fig. 1; see ‘Experimental procedure with harbour seals’, below).

To avoid secondary cueing, the experimenter hid behind a black curtain behind the harbour seals during the experiment. A camera (Logitech C270 HD Webcam, Logitech Europe S.A., Lausanne, Switzerland), connected to a laptop (Lenovo ThinkPad T420, Lenovo Deutschland GmbH, Stuttgart, Germany), was used to observe the behaviour of the harbour seals.

### Stimuli

The optic flow simulations were programmed and displayed in MatLab (version R2021b, The MathWorks^®^, Inc., Natick, MA, USA) and the associated Psychophysics Toolbox 3.0 (Brainard, 1997; Pelli, 1997; Kleiner et al., 2007).

In the experiment, we presented optic flow fields that simulated translational forward movements with a velocity of 2 m s^−1^, a typical swimming speed of a harbour seal (e.g. as assumed in the study of Bodson et al., 2007; Gläser et al., 2014; cf. Williams and Kooyman, 1985). The optic flow fields were generated by randomly placing white dots simulating the movement of a virtual observer through an otherwise empty virtual three-dimensional black space (photometric brightness of 0.01 cd m^−2^; LS-110, Minolta, Langenhagen, Germany) over a period of 29 s. Thus, each frame was a snapshot of the viewing frustum showing approximately 200 dots randomly positioned within a cubic volume extending 20 m in depth. During the simulated forward movement, dots dropped out of view at the front of the scene, and new dots were replenished in the far distance of the viewing frustum. Further, the dots did not change in size or contrast as they travelled through the virtual space in line with optic flow stimuli used to describe human optic flow perception (e.g. Warren et al., 1988). The diameter of the dots was 0.4 deg of visual angle and thus larger than the single target acuity of harbour seals (Sandow and Hanke, 2024).

Three optic flow environments were presented to the harbour seals. On the one hand, we displayed volume optic flow mimicking movement through a dot cloud as just described (for a schematic visualization, see Fig. 1B). On the other hand, we wanted to mimic surface optic flow by simulating a translational forward movement above or underneath a plane (for schematic visualizations, see Fig. 1C,D). For these optic flow environments, the dots were all arranged on a plane either 1.2 m below or 1.2 m above the harbour seal's point of view by adjusting the dots' height in the original volume optic flow field to a constant value of either −1.2 m or +1.2 m (Fig. 1C,D).

Irrespective of the optic flow environment, the FOE could be either to the left or to the right from the centre of the monitor, simulating a leftward or rightward movement. The FOE's position changed on the horizon of the plane (surface optic flow) or on the horizontal line transecting the centre of the monitor (volume optic flow), respectively. During basic task acquisition (see ‘Basic task acquisition’, below), the angle between the FOE and the vertical centre of the monitor, in the following called the heading angle, was set to 22 deg. For threshold determination (see ‘HAT determination’, below), we used the method of constant stimuli, presenting six heading angles to the harbour seal, namely 22, 18, 14, 10, 6 and 2 deg.

### Experimental procedure with harbour seals

The harbour seal was guided into the chamber and had to place its head into the metal hoop station in front of the black monitor. When the seal was positioned in the station, the experimenter closed the door and disappeared behind the curtain in the back of the experimental chamber. After a time interval of approximately 2 min, the first trial was started. In a two-alternative forced-choice task, the harbour seal had to indicate whether the simulated translational forward movement was to the left or right side of the vertical center of the monitor.

To indicate its response, the harbour seal had to touch one of the response targets with its snout. If it touched the response target on the side corresponding to the side of the monitor to which the simulated translational forward movement was directed, i.e. touching the left response target when presented with a leftward movement, and vice versa, the response was rewarded with fish. No reward was given after an incorrect response. After the harbour seal's response, the simulation was stopped and the monitor turned black. For a new trial to start, the harbour seal was sent back into the hoop station.

#### Basic task acquisition

Seals Nick and Luca were familiar with two-alternative forced-choice experiments (see ‘Experimental animals’, above). In contrast, we had to teach the general experimental procedure to the experimentally naive seal Miro as preparation for the optic flow experiment. This training included teaching Miro how to station in the experimental station, to pay attention to the monitor and to respond to stimuli presented on the monitor. For the last of these, the harbour seal was presented with a dot on one side of the monitor, either left or right, and had to move its snout to the left or right response target, respectively (Sandow and Hanke, 2024). Thus, all harbour seals participating in our study were familiar with the two-alternative forced-choice procedure before being presented with optic flow stimuli.

During the phase of basic task acquisition, one experimental session consisted of 30 trials. We presented optic flow stimuli simulating forward movements. Within a session, leftward or rightward movements were presented in pseudorandomized order after Gellermann (1933). We considered the harbour seals to have learnt the basic task upon reaching a learning criterion of ≥80% correct choices to be met in two consecutive sessions. For task consolidation, we performed sessions of overtraining, meaning that we continued to train after the harbour seals had already reached the learning criterion. After task consolidation, we started the sessions for threshold determination (see ‘HAT determination’, below).

In the first experimental phase, Nick and Miro were presented with the above-surface optic flow (see ‘Stimuli’, above), while Luca started with the volume optic flow (see ‘Stimuli’, above). We varied the type of optic flow environments during basic task acquisition between individuals to account for possible differences in the task acquisition between individuals depending on whether they were initially presented with the volume or above-surface optic flow simulation.

After the threshold determination for the first optic flow environment, we started the basic task acquisition phase for the second optic flow environment; thus, in the second experimental phase, Nick and Miro were presented with the volume optic flow, while Luca had to perform the task with above-surface optic flow.

After the second HAT determination, we conducted the experiment with the surface optic flow mimicking movement underneath a surface with all three harbour seals. This phase again consisted of task acquisition and HAT determination.

#### HAT determination

During HAT determination, we investigated the minimal angular deviation of the FOE from the centre of the simulation, corresponding to the centre of the monitor. For HAT determination, we followed the method of constant stimuli, meaning we pre-set six heading angles (see ‘Stimuli’, above) and presented each 30 times over the course of five sessions to the harbour seals to obtain a psychometric function (see Figs S1–S3) from which the HAT was determined.

The experimental sessions for HAT determination consisted of 36 trials, allowing the presentation of each of the six heading angles six times per experimental session. The side of the FOE was randomized after Gellermann (1933). For randomization of the six heading angles over the course of a session, we divided each session into three blocks of 12 trials each. Within each block, each of the six heading angles was displayed once on the left and once on the right side of the monitor. Within these blocks, the sequence of the heading angles was randomized using the RAND() function in Excel (Microsoft Office Professional Plus 2016 v.16.0.4266.1001, 2016 Microsoft Corporation, Redmond, WA, USA). This method aimed to mitigate potential differences in motivation across an experimental session, ensuring that any differences in motivation would affect the performance with respect to all instead of single heading angles.

After completion of the five sessions of threshold determination, the performance of the harbour seals in the 30 trials per heading angle was averaged in a psychometric function (see Figs S1–S3). The HAT, defined as the heading angle with which the harbour seal could determine the simulated heading at a performance of 75% correct choices, was then calculated from the psychometric function by linear interpolation of the performance of the last suprathreshold and first subthreshold heading angle, using Excel (Microsoft Office Professional Plus 2016 v.16.0.4266.1001, Microsoft Corporation). We collected several HATs subsequently (see Figs S1–S3). HAT determination was considered to be finalized once the harbour seal's performance did not improve over two consecutive HAT determinations, meaning the two HATs adopted the same values or the second was inferior to the first. The final HAT was then defined as the best HAT determined during the HAT determination phase, i.e. the best HAT from all determined HATs.

### HAT determination for human participants

HAT determination for human participants was conducted under the same experimental conditions and in the same experimental setup as with the harbour seals. Nevertheless, we slightly adjusted the experimental setup and procedure as follows. Instead of a hoop station, the human participants were instructed to place their chin on a chin station. The participants were told to communicate their judgement, leftward or rightward movement, by saying ‘left’ or ‘right’. No feedback regarding the correctness of their answers was provided. We skipped the basic task acquisition phase as we were able to explain the task to the participants. Further, we only determined one HAT for volume optic flow, above-surface optic flow and beneath-surface optic flow for each participant as usually done in human psychophysics (e.g. Warren et al., 1988; Duffy and Wurtz, 1993; Grigo and Lappe, 1998). The psychometric functions for HAT determination can be found in Fig. S4.

For HAT determination, we presented six heading angles of 14, 6, 2, 1, 0.5 and 0.1 deg. We used different heading angles for humans in comparison to those for the harbour seals because, based on results from previous studies (Van den Berg and Brenner, 1994; Warren and Hannon, 1990), human heading accuracy was anticipated to be superior to harbour seal heading accuracy.

## RESULTS

### Basic task acquisition

**Fig. 2.**
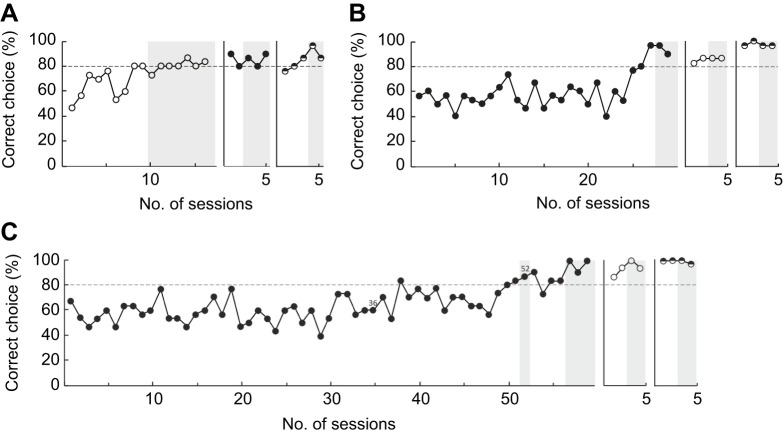
**Performance of the three harbour seals during basic task acquisition for volume optic flow and above- and beneath-surface optic flow.** The dashed line indicates the performance level of 80% correct choices the harbour seals had to reach/surpass in two consecutive sessions to fulfil the learning criterion. Areas with a grey shaded background mark sessions of overtraining. After basic task acquisition, we entered the heading accuracy threshold (HAT) determination phase. Once the HATs of one optic flow stimulus had been determined, we entered into the basic task acquisition phase for the next optic flow stimulus. (A) Learning curve of seal Luca. After Luca reached the learning criterion, we carried out seven sessions of overtraining with volume optic flow (open circles) before HAT determination. For above-surface optic flow (filled circles), we performed three sessions of overtraining and for beneath-surface optic flow (half-filled circles), we performed two sessions of overtraining after reaching the learning criterion. (B) Seal Nick was first presented with the above-surface optic flow. We carried out two sessions of overtraining for each of above-surface optic flow (filled circles), volume optic flow (open circles) and beneath-surface optic flow (half-filled circles). (C) The experimentally naive seal Miro took the longest to reach the learning criterion (sessions 36-52 with marked FOE, for details see Results, ‘Basic task acquisition’, above). After reaching the learning criterion with unmarked FOE, we performed three sessions of overtraining for above-surface optic flow (filled circles) before HAT determination. For both volume optic flow (open circles) and the subsequent beneath-surface optic flow (half-filled circles), we carried out two sessions of overtraining before HAT determination.

In the first experimental phase with harbour seals, Luca learnt to determine the simulated heading in nine sessions (Fig. 2A, Table 1); Nick reached the learning criterion within 27 sessions (Fig. 2B, Table 1); and Miro needed more than 50 sessions for basic task acquisition including a phase from session 36-51 in which a 0.6 deg large white dot was marking the FOE (Fig. 2C, Table 1). After one additional session with the marked FOE, seal Miro reached the learning criterion with unmarked FOE after four sessions.

**Table 1.** Overview of the various stages of heading accuracy threshold (HAT) determination with volume, above-surface and beneath-surface optic flow in harbour seals and humans

Subject	Stage	No. of stimuli	Heading angle (deg)	No. of sessions
Seal Luca	Volume optic flow			
	Basic task	1	22	9
	HAT determination	6	22/18/14/10/6/2	
	New HAT determination*	6	22/18/14/10/6/2	
	Surface optic flow (above)			
	Basic task	1	22	2
	HAT determination	6	22/18/14/10/6/2	
	Surface optic flow (underneath)			
	Basic task	1	22	3
	HAT determination	6	22/18/14/10/6/2	
Seal Nick	Surface optic flow (above)			
	Basic task	1	22	27
	HAT determination	6	22/18/14/10/6/2	
	Volume optic flow			
	Basic task	1	22	2
	HAT determination	6	22/18/14/10/6/2	
	Surface optic flow (underneath)			
	Basic task	1	22	2
	HAT determination	6	22/18/14/10/6/2	
Seal Miro	Surface optic flow (above)			
	Basic task	1	22	51
	HAT determination	6	22/18/14/10/6/2	
	Volume optic flow			
	Basic task	1	22	2
	HAT determination	6	22/18/14/10/6/2	
	Surface optic flow (underneath)			
	Basic task	1	22	2
	HAT determination	6	22/18/14/10/6/2	
Humans	Volume optic flow			
	HAT determination	6	14/6/2/1/0.5/0.1	
	Surface optic flow (above)			
	HAT determination	6	14/6/2/1/0.5/0.1	
	Surface optic flow (underneath)			
	HAT determination	6	14/6/2/1/0.5/0.1	

For all stages, the number of stimuli (i.e. the number of heading angles per session), the heading angle(s) (meaning the angle between the FOE and the vertical center of the monitor in degrees) and the number of sessions needed to reach the learning criterion (≥80% correct choices in two consecutive sessions) including the two sessions in which the learning criterion was fulfilled are indicated. *With harbour seal Luca, we carried out a second HAT determination phase some months after the first HAT determination phase, as the first HAT determined clearly deviated from the HATs of the other harbour seals and as we had clear signs of the first HAT determination having been influenced by low motivation of the harbour seal over the summer months.

In the second experimental phase, all harbour seals transferred the task immediately, meaning that they reached the learning criterion within the minimally required two consecutive sessions, to the new optic flow environment (Fig. 2, Table 1).

In the third experimental phase, Nick and Miro again only needed two sessions to reach the learning criterion, while Luca needed three sessions (Fig. 2, Table 1).

### HAT

#### Harbour seals

**Fig. 3.**
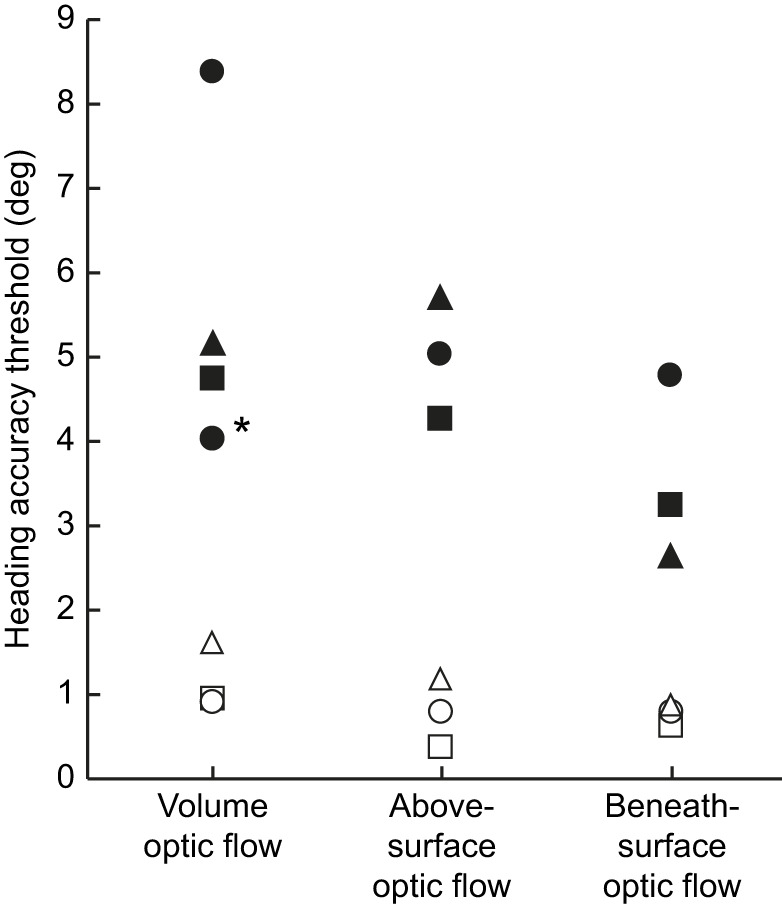
**Determined HATs for volume, above-surface and beneath-surface optic flow for the three harbour seals and three human participants.** Data are for seals Luca (filled circles), Nick (filled squares) and Miro (filled triangles) and the human participants H1 (open squares), H2 (open triangles) and H3 (open circles). Please note that we collected another HAT (marked with an asterisk) for volume optic flow with seal Luca some months after the first HAT determination (see Materials and Methods and Results ‘Harbour seals’).

For a simulation of a translational forward movement through a dot cloud, inducing volume optic flow, Luca, Nick and Miro determined the simulated heading with a HAT of 8.33, 4.75 and 5.09 deg, respectively (Fig. 3). As Luca's HAT (Fig. 3) deviated strongly from the HATs of the other harbour seals, and as this harbour seal showed signs of low motivation during the time of data collection taking place in the summer months, we replicated the HAT some months later. In this second HAT determination phase, Luca was able to determine the simulated heading with a threshold of 4.00 deg (see Fig. 3). Using this HAT to calculate a mean HAT for volume optic flow for all three harbour seals, the mean (±s.d.) HAT amounted to 4.61±0.56 deg (*N*=3).

During a simulated translational forward movement above a surface, the harbour seals needed at least an angular difference of 5.00 deg for Luca, 4.20 deg for Nick and 5.67 deg for Miro (Fig. 3) between the FOE and the vertical centre of the monitor to determine the simulated heading at threshold performance. These individual HATs resulted in a mean HAT of 4.96±0.74 deg (*N*=3).

For a simulation of optic flow elicited when swimming underneath a surface, Luca determined the simulated heading with a HAT of 4.83 deg (Fig. 3). Nick reached a HAT of 3.25 deg and Miro needed an angular difference of 2.67 deg between the FOE and the vertical centre of the monitor to determine the heading (Fig. 3). The individual HATs resulted in a mean HAT of 3.58±1.12 deg (*N*=3).

#### Human participants

During simulation of movement through a dot cloud, inducing volume optic flow, the human participants indicated the simulated heading with a HAT of 0.96 deg (H1), 1.54 deg (H2) and 0.95 deg (H3; Fig. 3), resulting in a mean HAT of 1.15±0.34 deg (*N*=3).

For simulation of a translational forward movement above a surface, the human participants H1, H2 and H3 reached HATs of 0.36, 1.08 and 0.88 deg, respectively (Fig. 3), resulting in a mean HAT of 0.77±0.37 deg (*N*=3).

During simulation of beneath-surface optic flow, the human participants reached HATs of 0.68 deg (H1), 0.89 deg (H2) and 0.88 deg (H3; Fig. 3), resulting in a mean HAT of 0.82±0.12 deg (*N*=3).

## DISCUSSION

Our results indicate that harbour seals can determine the direction of self-motion from surface optic flow as well as volume optic flow. Based on our results of aerial and underwater optic flow perception (this study; Gläser et al., 2014), we expect harbour seals to be able to rely on optic flow information under any circumstances in which they move through an environment with enough light for vision available and with at least some objects with enough contrast to allow motion perception. This richly available optic information can be used for the determination of (deviations of) heading (this study; Gläser et al., 2014), and future studies will test whether harbour seals can use optic flow for additional behaviours, e.g. distance estimation, collision avoidance and control of swimming speed. Control of swimming speed might be particularly interesting to look at as one publication on optic flow perception in zebrafish (Scholtyssek et al., 2014) suggested the fish, unlike flying insects such as honeybees (e.g. Srinivasan, 2011), do not rely on optic flow for the control of movement velocity.

Our findings stress the importance of considering the effect of movement on marine mammal sensory perception. So far, this topic has hardly been considered regarding marine mammals (but see for example Bodson et al., 2007; Hanke et al., 2024); previous studies on sensory perception in marine mammals have primarily considered the performance of stationary animals. That approach contrasts with the fact that in most instances an animal is moving when performing complex behaviours. Regarding vision, the consideration of movement and thus of optic flow results in a paradigm shift. Therefore, it is of ultimate importance to consider the effect of movement not only on vision but also on sensory perception in general; researchers need to focus on how sensory stimuli change over time when an animal is in motion.

Although the HATs did not differ strongly between harbour seal individuals in our experiment, the rate of basic task acquisition differed inter-individually in our study. Most likely, this different acquisition rate is a reflection of the different experimental histories of the harbour seals. Luca, who showed the fastest rate of acquisition, had already participated in numerous scientific experiments including optic stimuli (e.g. Heinrich et al., 2016; Sandow and Hanke, 2024; Scholtyssek et al., 2008). In absolute terms, Luca's rate of acquisition, and that of harbour seal Malte from a previous study (Gläser et al., 2014; rate of acquisition: F.D.H., personal observation), was even faster than that in past studies involving simple optic stimuli (F.D.H., personal experience). Second, Nick had less experience with optic stimuli (but see Mauck and Dehnhardt, 2005; Sandow and Hanke, 2024) and performed slightly worse in comparison to Luca. Finally, Miro, who was experimentally naive, needed almost six times as many sessions for basic task acquisition compared with Luca and a phase with marked FOE. Nevertheless, Miro learnt the task and finally showed a performance equal to that of the more versed harbour seals. Thus, while experience seems to affect the rate of learning, it does not have an effect on the ability of harbour seals to determine their heading from optic flow.

From our current results, we cannot postulate a better access of harbour seals to volume versus surface optic flow or vice versa. Regarding the acquisition rate, seal Luca from the current study and seal Malte from the first optic flow study (Gläser et al., 2014; rate of acquisition: F.D.H., personal observation) learnt to determine (deviations of) their heading from volume optic flow very quickly, faster than the other harbour seals presented with surface optic flow. However, it is hard to separate the rate of acquisition with respect to an optic flow scenario from the experimental experience. Luca and Malte had already participated in challenging visual experiments (see Materials and Methods, ‘Experimental animals’ and, for example, Mauck and Dehnhardt, 2005), which might have facilitated the learning process with optic flow stimuli. Regarding the HATs, they generally did not differ strongly between optic flow scenarios. Although the observed tendencies should not be overemphasized, we observed that the HATs from volume and beneath-surface optic flow tended to be slightly superior to the HATs from above-surface optic flow. In line with the model of Koenderink and van Doorn (1987), one explanation for this tendency could be that the two optic flow scenarios which resulted in a slightly superior HAT performance fill larger parts of the visual field of harbour seals (Hanke et al., 2006) than above-surface optic flow, with the latter only filling a small part of the restricted ventral visual field of harbour seals. In conclusion, harbour seals generally seem to have good access to surface as well as volume optic flow, which allows them to rely on their optic flow system very flexibly and irrespective of which optic flow environment they experience.

The harbour seals' heading accuracy was comparable, but slightly inferior to the heading accuracy of humans and Rhesus macaques (e.g. this study; Warren et al., 1988; Britten and van Wezel, 2002). As our harbour seals ultimately worked with a high level of motivation/attention, we repeatedly assessed the HATs for each individual, and since the HATs determined for the three harbour seals were very similar, the slight superiority of the humans’ versus harbour seals’ HAT can most likely be ascribed to differences in where/how optic flow stimuli are being processed. The neuronal correlates of optic flow perception were well described for primates (Britten and van Wezel, 1998; Lappe et al., 1996; Gu et al., 2010), but still need to be determined for harbour seals.

The HATs we determined with our harbour seals are slightly inferior to our results with humans obtained under highly similar experimental conditions. While we can compare our results for harbour seals and humans directly, results of previous psychophysical experiments on optic flow perception in humans and primates (e.g. Warren et al., 1988; Warren and Hannon, 1990; Van den Berg and Brenner, 1994; Lappe et al., 1996; Britten and van Wezel, 2002) are more difficult to compare because of the methodological differences of these studies. One apparent methodological difference is that many of these studies used reference objects, either fixation points (e.g. Britten and van Wezel, 2002) or target lines (e.g. Warren et al., 1988; Warren and Hannon, 1990), to determine heading accuracy. A reference object was also used in the first harbour seal optic flow study (Gläser et al., 2014); the harbour seal had to determine deviations of a cross from a simulated heading. As the single threshold determined by Gläser et al. (2014) is superior to the thresholds determined in our current study and as this previous threshold compares well with human psychophysical thresholds, future research could expand this line of research assessing whether the insertion of a reference point indeed improves performance in a heading determination task in harbour seals.

To start with an in-depth characterization of optic flow perception in harbour seals, we designed the schematic optic stimuli as in previous human optic flow experiments (Warren et al., 1988): (1) the dots did not change in size or contrast when moving through the virtual space, (2) optic flow fields included only frontal optic flow and (3) maximum viewing distance was set to a fixed value of 20 m. Although these stimuli lacked some natural optic flow characteristics, they contained all relevant information of the frontal optic flow field and for heading determination. Thus, we would not expect the HATs to be different when testing heading accuracy with more naturalistic optic flow stimuli as all vectors regardless of angular size as well as contrast as measures for distance to the harbour seal provide the necessary heading information. Nevertheless, future experiments particularly on underwater optic flow perception (see below) will also have to consider more ecologically relevant scenarios.

From (1) the relatively fast acquisition of the rather complex optic flow stimuli, (2) the fact that even an inexperienced harbour seal learnt the task within a decent amount of time and reached HATs comparable to those of the other harbour seals tested in this study and (3) the fact that the harbour seals easily transferred between optic flow scenarios, even with a break of 1 year between experimental phase 2 and 3, we deduce that optic flow can be a strong and easily discernible stimulus for harbour seals providing evidence for the significance of optic flow for harbour seals in their natural environment. We would thus anticipate that harbour seals will be able to perform even more complex behaviours based on optic flow information, an aspect that awaits future experimentation.

While this study described aerial optic flow perception, future research on harbour seal optic flow perception should concentrate on the specifics of underwater optic flow, which are largely unstudied (but see Gläser et al., 2014; Scholtyssek et al., 2014). To give an example, when thinking about movement through a turbid environment, particles dissolved in the water column are not rigid objects. The particles may be drifting as a result of sea currents. Whether and how aquatic animals can cope with this condition is a topic for future research. Altogether we expect investigations on optic flow perception in aquatic animals to extend our understanding of optic flow perception in general. We expect these studies to have the potential to revolutionize our understanding of vision in aquatic animals and regarding complex behaviours in particular.

## Supplementary Material

10.1242/jexbio.250168_sup1 Supplementary information

Dataset 1. Above surface optic flow.

Dataset 2. Volume optic flow.

Dataset 3. Below surface optic flow.

## References

[JEB250168C1] Baird, E., Kreiss, E., Wcislo, W., Warrant, E. and Dacke, M. (2011). Nocturnal insects use optic flow for flight control. *Biol. Lett.* 7, 499-501. 10.1098/rsbl.2010.1205 21307047 PMC3130226

[JEB250168C2] Barron, A. and Srinivasan, M. V. (2006). Visual regulation of ground speed and headwind compensation in freely flying honey bees (*Apis mellifera L*.). *J. Exp. Biol.* 209, 978-984. 10.1242/jeb.02085 16481586

[JEB250168C3] Bodson, A., Miersch, L., Mauck, B. and Dehnhardt, G. (2007). Underwater auditory localization by a swimming harbour seal (*Phoca vitulina*). *J. Acoust. Soc. Am.* 120, 1550-1557. 10.1121/1.2221532 17004477

[JEB250168C4] Brainard, D. H. (1997). The psychophysics toolbox. *Spat. Vis.* 10, 433-436. 10.1163/156856897X00357 9176952

[JEB250168C40] Britten, K. H. and van Wezel, R. J. A. (1998). Electrical microstimulation of cortical area MST biases heading perception in monkeys. *Nature Neurosci.* 1, 59-63. 10.1038/259 10195110

[JEB250168C5] Britten, K. H. and Van Wezel, R. J. A. (2002). Area MST and heading perception in macaque monkeys. *Cerebral Cortex* 12, 692-701. 10.1093/cercor/12.7.692 12050081

[JEB250168C6] Dehnhardt, G., Hanke, W., Wieskotten, S., Krüger, Y. and Miersch, L. (2014). Hydrodynamic perception in seals and sea lions. In *Flow Sensing in Air and Water* (ed. H. Bleckmann). Springer.

[JEB250168C7] Duffy, C. J. and Wurtz, R. H. (1993). An illusory transformation of optic flow fields. *Vis. Res.* 33, 1481-1490. 10.1016/0042-6989(93)90141-I 8351820

[JEB250168C8] Esch, H. E. and Burns, J. E. (1995). Honeybees use optic flow to measure the distance of a food source. *Naturwissenschaften* 82, 38-40. 10.1007/BF01167870

[JEB250168C9] Gellermann, L. W. (1933). Chance orders of alternating stimuli in visual discrimination experiments. *Pedagog. Semin. J. Genet. Psychol.* 42, 206-208. 10.1080/08856559.1933.10534237

[JEB250168C10] Geurten, B. R. H., Niesterok, B., Dehnhardt, G. and Hanke, F. D. (2017). Saccadic movement strategy in a semiaquatic species - the harbour seal (*Phoca vitulina*). *J. Exp. Biol.* 220, 1503-1508. 10.1242/jeb.150763 28167803

[JEB250168C11] Gibson, J. J. (1950). *The Perception of the Visual World*. Boston: Houghton Mifflin. 10.1126/science.113.2940.535.a

[JEB250168C12] Gläser, N., Mauck, B., Kandil, F. I., Lappe, M., Dehnhardt, G. and Hanke, F. D. (2014). Harbour seals (*Phoca vitulina*) can perceive optic flow under water. *PLoS ONE* 9, Article e103555. 10.1371/journal.pone.0103555 25058490 PMC4110048

[JEB250168C13] Grigo, A. and Lappe, M. (1998). An analysis of heading towards a wall. In *Vision and Action* (ed. L. R. Harris and M. Jensin). The Press Syndicate of the University of Cambridge.

[JEB250168C14] Gu, Y., Fetsch, C. R., Adeyemo, B., Deangelis, G. C. and Angelaki, D. E. (2010). Decoding of MSTd population activity accounts for variations in the precision of heading perception. *Neuron* 66, 596-609. 10.1016/j.neuron.2010.04.026 20510863 PMC2889617

[JEB250168C41] Hanke, F. D., Hanke, W., Hoffmann, K.-P. and Dehnhardt, G. (2008). Optokinetic nystagmus in harbor seals (*Phoca vitulina*). *Vision Res.* 48, 304-315. 10.1016/j.visres.2007.11.012 18160091

[JEB250168C16] Hanke, W., Römer, R. and Dehnhardt, G. (2006). Visual fields and eye movements in a harbor seal (*Phoca vitulin*a). *Vis. Res.* 46, 2804-2814. 10.1016/j.visres.2006.02.004 16564067

[JEB250168C18] Hanke, F. D., Hanke, W., Scholtyssek, C. and Dehnhardt, G. (2009). Basic mechanisms in pinniped vision. *Exp. Brain Res.* 199, 299-311. 10.1007/s00221-009-1793-6 19396435

[JEB250168C19] Hanke, F. D., Mooney, T. A. and Janik, V. M. (2024). Sensory physiology in delphinids. In *The Physiology of Dolphins* (ed. S. Hooker and A. Fahlmann). Elsevier.

[JEB250168C20] Heinrich, T., Dehnhardt, G. and Hanke, F. D. (2016). Harbour seals (*Phoca vitulina*) are able to time precisely. *Anim. Cogn.* 19, 1133-1142. 10.1007/s10071-016-1020-3 27496205

[JEB250168C21] Heinrich, T., Ravignani, A. and Hanke, F. D. (2020). Visual timing abilities of a harbour seal (*Phoca vitulina*) and a South African fur seal (*Arctocephalus pusillus pusillus*) for sub- and supra-second time intervals. *Anim. Cogn.* 23, 851-859. 10.1007/s10071-020-01390-3 32388781 PMC7415748

[JEB250168C22] Kleiner, M., Brainard, D. H., Pelli, D., Ingling, A., Murray, R. and Broussard, C. (2007). What's new in Psychtoolbox-3. *Perception* 36, 1-16.

[JEB250168C23] Koenderink, J. J. (1986). Optic flow. *Vis. Res.* 26, 161-180. 10.1016/0042-6989(86)90078-7 3716209

[JEB250168C24] Koenderink, J. J. and Van Doorn, A. J. (1987). Facts on optic flow. *Biol. Cybern.* 56, 247-254. 10.1007/BF00365219 3607100

[JEB250168C25] Lappe, M., Bremmer, F., Pekel, M., Thiele, A. and Hoffmann, K.-P. (1996). Optic flow processing in Monkey STS: a theoretical and experimental approach. *J. Neurol.* 16, 6265-6285. 10.1523/JNEUROSCI.16-19-06265.1996 PMC6579186 8815907

[JEB250168C26] Maaß, E. and Hanke, F. D. (2021). Distance estimation in reproduction tasks in a harbour seal (*Phoca vitulina*). *Water* 13, 938. 10.3390/w13070938

[JEB250168C27] Mauck, B. and Dehnhardt, G. (2005). Identity concept formation during visual multiple-choice matching in a harbor seal (*Phoca vitulina*). *Learn. Behav.* 33, 428-436. 10.3758/BF03193181 16573213

[JEB250168C28] Pelli, D. G. (1997). The VideoToolbox software for visual psychophysics: transforming numbers into movies. *Spat. Vis.* 10, 437-442. 10.1163/156856897X00366 9176953

[JEB250168C29] Ronacher, B. and Wehner, R. (1995). Desert ants *Cataglyphis fortis* use self-induced optic flow to measure distances travelled. *J. Comp. Physiol. A* 177, 21-27. 10.1007/BF00243395

[JEB250168C30] Sandow, L.-M. and Hanke, F. D. (2024). Aerial single target acuity in harbour seals (*Phoca vitulina*). *Vis. Res.* 218, 108389. 10.1016/j.visres.2024.108389 38531191

[JEB250168C31] Scholtyssek, C., Kelber, A. and Dehnhardt, G. (2008). Brightness discrimination in the harbour seal (*Phoca vitulina*). *Vis. Res.* 48, 96-103. 10.1016/j.visres.2007.10.012 18078667

[JEB250168C32] Scholtyssek, C., Dacke, M., Kröger, R. H. H. and Baird, E. (2014). Control of self-motion in dynamic fluids: fish do it differently from bees. *Biol. Lett.* 10, 2010279. 10.1098/rsbl.2014.0279 PMC4046384 24872463

[JEB250168C33] Srinivasan, M. V. (2011). Honeybees as a model for the study of visually guided flight, navigation, and biologically inspired robotics. *Physiol. Rev.* 91, 413-460. 10.1152/physrev.00005.2010 21527730

[JEB250168C34] Sun, H. J., Carey, D. P. and Goodale, M. A. (1992). A mammalian model of optic-flow utilization in the control of locomotion. *Exp. Brain Res.* 9, 91. 10.1007/BF00230026 1301371

[JEB250168C35] Van Den Berg, A. and Brenner, E. (1994). Humans combine the optic flow with static depth cues for robust perception of heading. *Vis. Res.* 34, 2153-2167. 10.1016/0042-6989(94)90324-7 7941412

[JEB250168C36] Warren, W. H. (2008). Optic flow. In *The Senses: A Comprehensive Reference* (ed. A. I. Basbaum, A. Kaneko, G. M. Shepherd and G. Westheimer), Vol. 2, pp. 219-230. Academic Press. 10.1016/B978-012370880-9.00311-X

[JEB250168C37] Warren, W. H. and Hannon, D. J. (1990). Eye movements and optical flow. *J. Opt. Soc. Am. A* 7, 160-169. 10.1364/JOSAA.7.000160 2299447

[JEB250168C38] Warren, W. H., Morris, M. W. and Kalish, M. (1988). Perception of translational heading from optical flow. *J. Exp. Psychol. Hum. Percept. Perform.* 14, 646-660. 10.1037/0096-1523.14.4.646 2974874

[JEB250168C39] Weiffen, B., Mauck, B., Dehnhardt, G. and Hanke, F. D. (2014). Sensitivity of a harbor seal (*Phoca vitulina*) to coherent visual motion in random dot displays. *SpringerPlus* 3, 1-10. 10.1186/2193-1801-3-688 25520911 PMC4258534

[JEB250168C42] Williams, T. M. and Kooyman, G. L. (1985). Swimming performance and hydrodynamic characteristics of harbor seals *Phoca vitulina.* *Physiol. Zool.* 58, 576-589.

