# Supplementary material for: Optic flow, a rich source of optic information for harbour seals (Phoca vitulina)
Source: J Exp Biol. 2025 May 29;228(10):jeb250168. doi: 10.1242/jeb.250168 (PMC12148030; doi:10.1242/jeb.250168)
Supplement: Supplementary information [file jexbio-228-250168-s1.pdf]

Supplementary Materials and Methods

Figure S1 - S3

We determined heading accuracy thresholds (HAT) for volume, above surface and beneath surface optic flow. For HAT determination the animals had to perform five sessions á 36 trials. Within these five sessions each heading angle was presented 30 times in total. The HAT, defined as the heading angle with which the harbour seal could determine the simulated heading at a performance of 75% correct choices in 60 cm distance, was then calculated by linear interpolation of the performance of the last supra- and first subthreshold heading angle. We determined HATs until the harbour seals threshold did not further improve over two consecutive HAT determinations. The best HAT was defined as the final HAT and is reported in the results section in the manuscript.

Figure S4

We determined HATs for the same optic flow simulations as with the harbour seals in three human participants. For HAT determination the participants had to perform five sessions á 36 trials. Within these five sessions each heading angle was presented 30 times in total. The HAT, defined as the heading angle with which the participants could determine the simulated heading at a performance of 75% correct choices in 60 cm distance, was then calculated by linear interpolation of the performance of the last supra- and first subthreshold heading angle. We determined one HAT for each optic flow simulation, which is reported in the results section in the manuscript, because we expected the participants to immediately show their best performance.

Please note: the data presented in the manuscript reflects the complete HAT data sets we obtained for H1-H3. Two additional participants were recorded with volume and above surface optic flow. The data from these participants is not shown in Fig. 3 in the main manuscript since no data from the beneath surface optic flow condition was available. Figure S4 however includes the psychometric functions including the HATs for volume optic flow and for above surface optic flow for these two additional human participants H4 and H5.

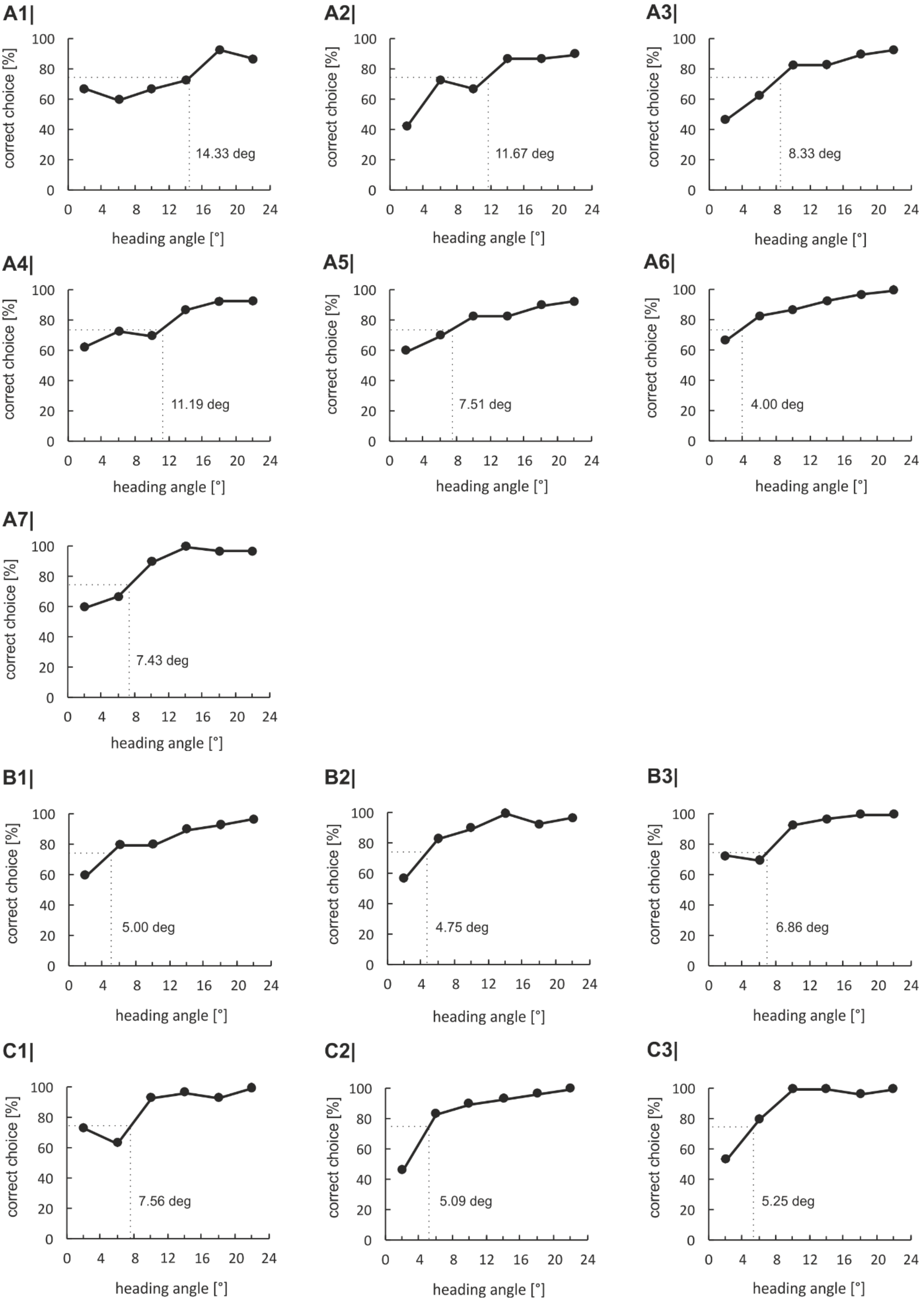
A1|
14.33 deg
A2|
11.67 deg
A3|
8.33 deg
A4|
11.19 deg
A5|
7.51 deg
A6|
4.00 deg
A7|
7.43 deg
B1|
5.00 deg
B2|
4.75 deg
B3|
6.86 deg
C1|
7.56 deg
C2|
5.09 deg
C3|
5.25 deg
correct choice [%]
heading angle [°]
0
20
40
60
80
100
0
4
8
12
16
20
24

Fig. S1. Psychometric functions, shown as the average performance of five sessions (in percent) for the six tested heading angles (in degree), for the calculation of the volume optic flow HAT of seal Luca (A), seal Nick (B) and seal Miro (C). The horizontal dotted line indicates the 75% performance and the vertical dotted lines represent the 75% HAT. The corresponding HAT value in degree is shown next to the vertical line. With seal Luca, we determined seven HATs (A1-A7), until his threshold did not further improve in two consecutive HAT determination. With seal Nick (B1-B3) and Miro (C1-C3) we determined three HATs, until the threshold did not further improve in two consecutive HAT determinations.

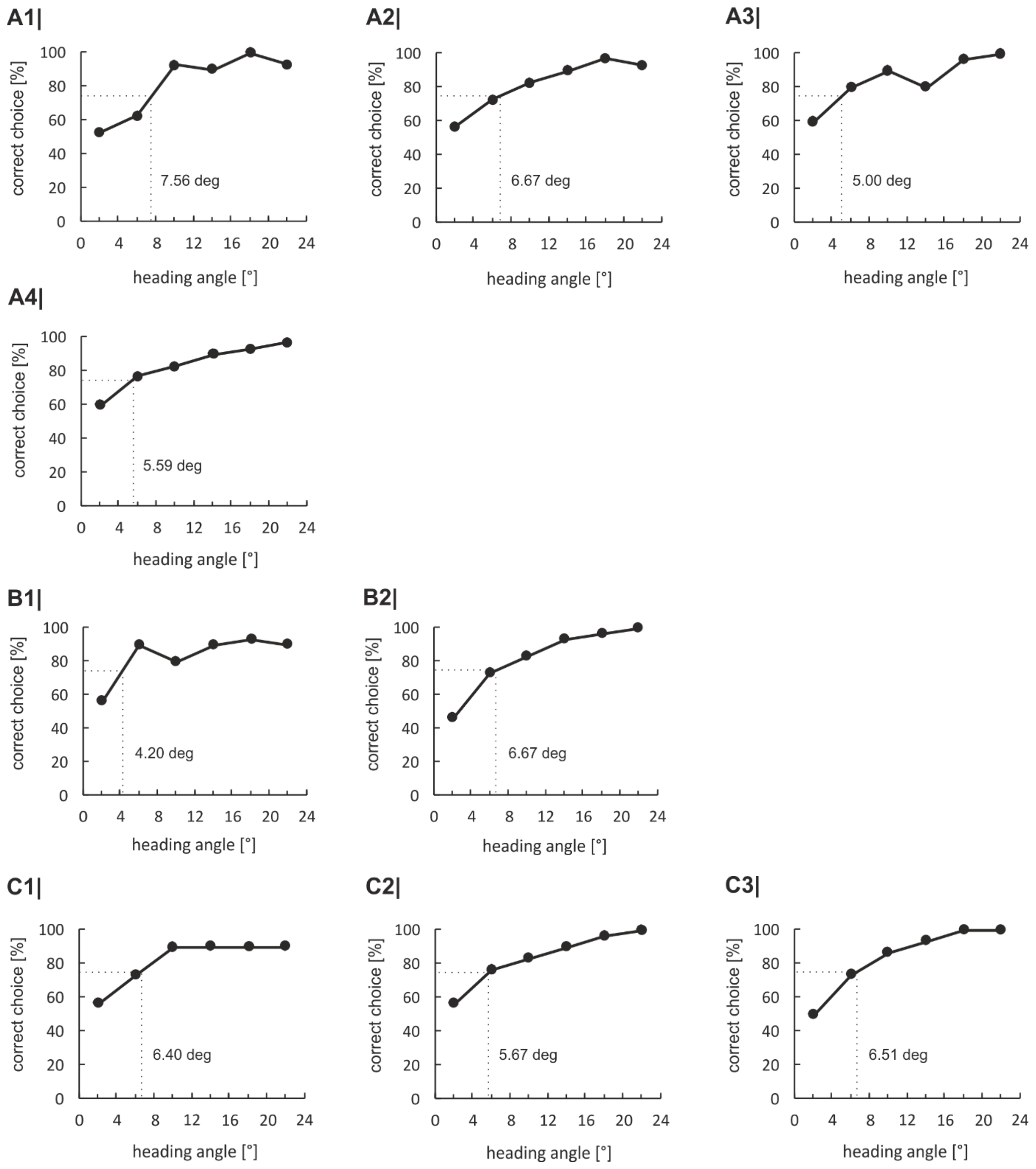

Fig. S2. Psychometric functions, shown as the average performance of five sessions (in percent) for the six tested heading angles (in degree), for the calculation of the above surface optic flow HAT of seal Luca (A), seal Nick (B) and seal Miro (C). The horizontal dotted line indicates the 75% performance and the vertical dotted lines represent the 75% HAT. The corresponding HAT value in degree is shown next to the vertical line. We determined four HATs with seal Luca (A1-A4), two HATs with seal Nick (B1-B2) and three HATs with seal Miro (C1-C3) until the criterion is reached which states that the HAT no longer improves in two consecutive HAT determinations.

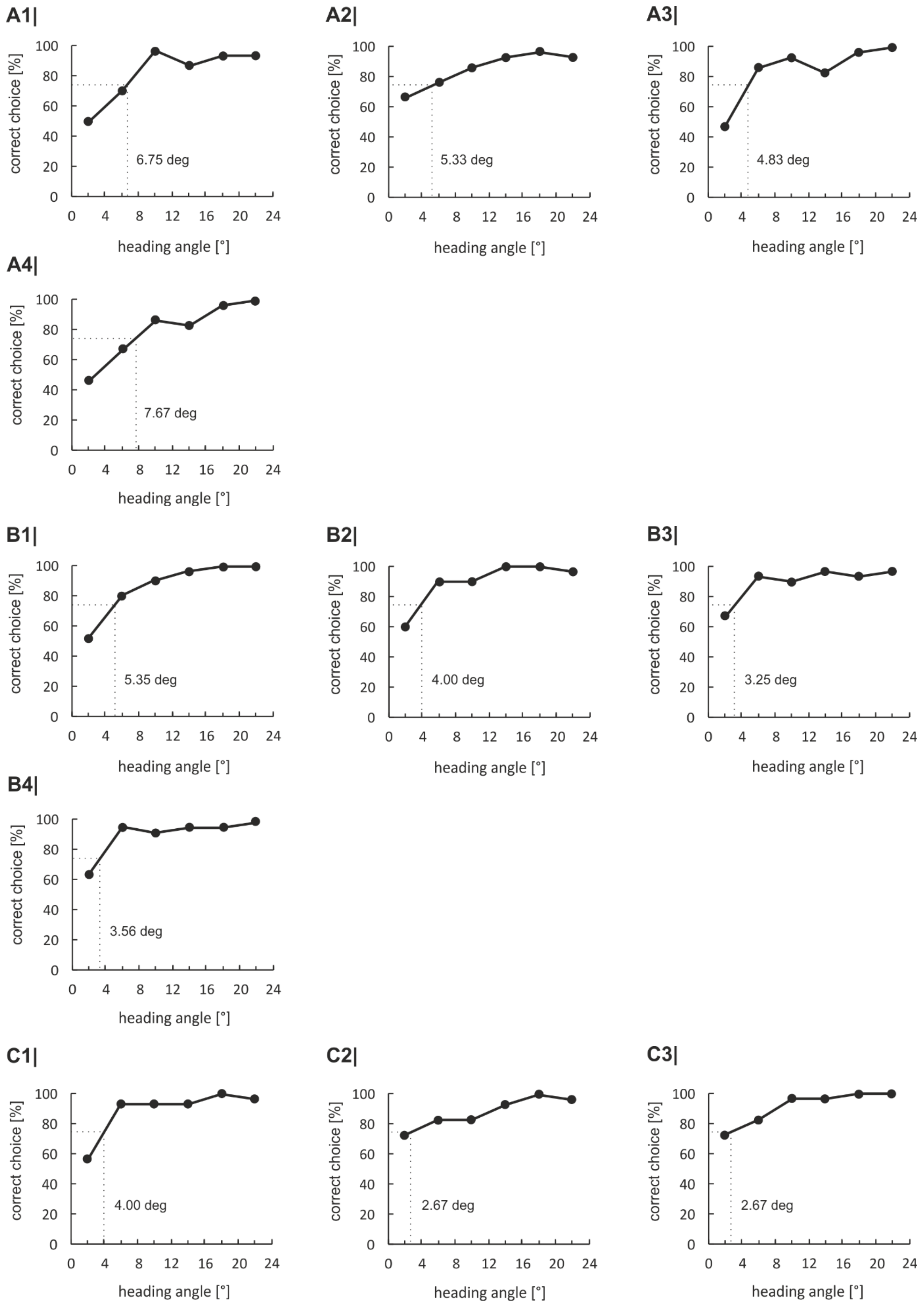
A1|
6.75 deg
A2|
5.33 deg
A3|
4.83 deg
A4|
7.67 deg
B1|
5.35 deg
B2|
4.00 deg
B3|
3.25 deg
B4|
3.56 deg
C1|
4.00 deg
C2|
2.67 deg
C3|
2.67 deg
correct choice [%]
heading angle [°]
0
20
40
60
80
100
0
4
8
12
16
20
24

Fig. S3. Psychometric functions, shown as the average performance of five sessions (in percent) for the six tested heading angles (in degree), for the calculation of the beneath surface optic flow HAT of seal Luca (A), seal Nick (B) and seal Miro (C). The horizontal dotted line indicates the 75% performance and the vertical dotted lines represent the 75% HAT. The corresponding HAT value in degree is shown next to the vertical line. With seal Luca (A1-A4) and seal Nick (B1-B4) we determined four HATs until the criterion is reached which states that the HAT no longer improves in two consecutive HAT determinations. With seal Miro, we determined three HATs to reach the criterion (C1-C3).

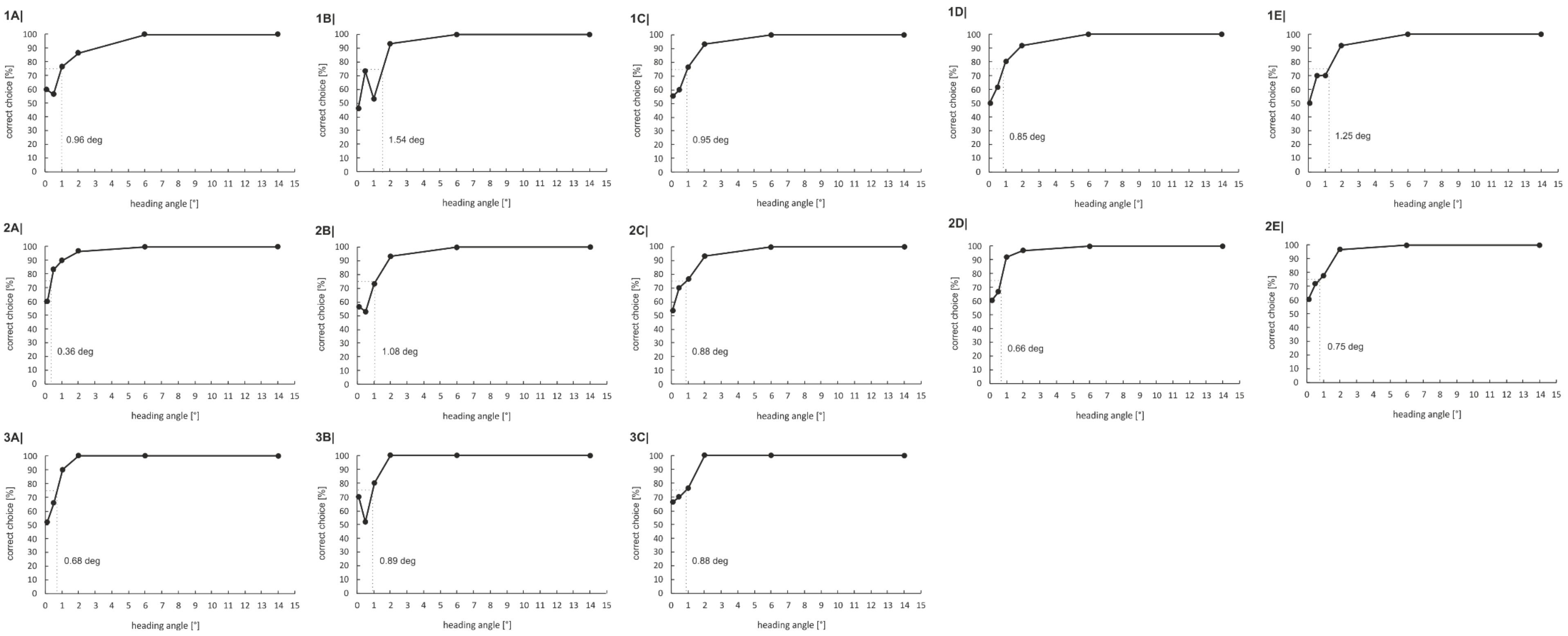

Fig. S4. Psychometric functions for 1| volume optic flow, 2| above surface and 3| beneath surface optic flow, shown as the average performance of five sessions (in percent) for the six tested heading angles, for the calculation of the HAT for the human participants (full datasets presented in the manuscript: A – H1, B – H2, C – H3; partial dataset not presented in the main manuscript, see note above: D – H4, E – H5). The horizontal dotted line indicates the 75% performance and the vertical dotted lines represents the 75% HAT. The corresponding HAT value in degree is shown next to the vertical line.

Journal of Experimental Biology • Supplementary information

Dataset 1. Above surface optic flow.

Available for download at
https://journals.biologists.com/jeb/article-lookup/doi/10.1242/jeb.250168#supplementary-data

Dataset 2. Volume optic flow.

Available for download at
https://journals.biologists.com/jeb/article-lookup/doi/10.1242/jeb.250168#supplementary-data

Dataset 3. Beneath surface optic flow.

Available for download at
https://journals.biologists.com/jeb/article-lookup/doi/10.1242/jeb.250168#supplementary-data
